# Association between thermal inversion and cognitive trajectories among middle-aged and older adults in CHARLS: A latent class trajectory analysis

**DOI:** 10.1371/journal.pone.0335902

**Published:** 2025-11-11

**Authors:** Kai Liu, Jing Wang, Junru Wang, Yue Yang, Yali Wang, Jiahui Zhang, Xiaojun Ma, Zhuoyuan Li, Ping Chen, Jiangping Li

**Affiliations:** 1 Department of Epidemiology and Health Statistics, School of public health, Ningxia Medical University, Yinchuan, Ningxia Hui Autonomous Region, China; 2 Medical Experiment Center, General Hospital of Ningxia Medical University, Yinchuan, China; 3 Ningxia Key Laboratory of Clinical and Pathogenic Microbiology, General Hospital of Ningxia Medical University, Yinchuan, China; 4 Key Laboratory of Environmental Factors and Chronic Disease Control, Ningxia Medical University, Yinchuan, Ningxia Hui Autonomous Region, China; University of Thessaly Faculty of Medicine: Panepistemio Thessalias Tmema Iatrikes, GREECE

## Abstract

**Background:**

Previous studies have demonstrated that cognitive decline is related to meteorological conditions, but most of them focus on air pollutants rather than thermal inversion (TI). The impact of TI on cognitive function remains unclear. Therefore, this study aims to explore the role of TI in the changes of cognitive function trajectories.

**Methods:**

This study included 5,762 participants aged 45 years and older from China Health and Retirement Longitudinal Study (CHARLS) between 2011−2015. The latent class trajectory model (LCTM) was used to fit population trajectories of cognitive development. The TI data were obtained from NASA’s MERRA-2 dataset, which were totaled by comparing temperatures of atmosphere. The multinomial logistic regression model and restricted cubic spline (RCS) were used to assess the relationship between TI and cognition, the primary outcome was class membership in cognitive trajectories estimated by LCTM.

**Results:**

There were three trajectories of cognitive development in 5,762 participants, which were categorized into three trajectories: U-shaped (decline then improvement), Slowly decline and N-shaped (improvement then decline). In the Slowly decline class, the proportion of individuals exposed to moderate and high levels of TI was the highest. After adjusting for covariates, at medium TI exposure, the odds of being classified into the Slowly decline trajectory versus the U-shaped trajectory were 26.9% higher (OR=1.269, 95% CI = 1.054 ~ 1.528, *P* = 0.012), at high exposure, 47.8% higher (OR=1.478, 95% CI = 1.152 ~ 1.895, *P* = 0.002).

**Conclusion:**

Higher TI exposure was associated with trajectory membership patterns indicative of worse cognition, strategies that reduce TI exposure may support healthier cognitive trajectories.

## 1. Introduction

Cognitive decline is prevalent among older adults, and in some patients, it may even develop into dementia [1]. With World Health Organization (WHO) surveys suggesting that there are 50 million people living with dementia globally today [2], and that by 2050, this number will rise to 113 million [3]. While WHO data may be less accurate for some countries or regions lacking corresponding statistical information, the data it compiles still reflects that the situation regarding dementia is extremely serious in China and other countries with relatively well-developed healthcare systems. China has nearly a quarter of the world’s dementia patients [4]. The proportion of elderly people in China is expected to reach 20% by 2037 [5], which will further exacerbate the burden of dementia in China. With the total annual cost of dementia within China increasing from $0.9 billion in 1990 to $47.2 billion in 2010, it is expected to reach 114.2 billion U.S. dollars by 2030 [6]. And the prevalence of dementia thought to be doubling every five years [7]. The surge in the elderly population has made cognitive decline an issue that cannot be ignored. Since there is no effective cure for dementia [1], it is necessary to study the risk factors associated with dementia and to prevent them [8].

Previous research on cognitive decline has focused on behavioral habits [9], weather conditions and related pollutants [10], the effects of thermal inversion (TI) on cognitive abilities as an atmospheric anomaly is not yet available. In general, atmospheric temperature decreases incrementally with altitude. The defining characteristic of TI lies in the anomalous elevation of atmospheric temperature with increasing altitude [11]. Furthermore, studies have shown that TI in the near-surface atmosphere has a greater impact on human health [12]. This phenomenon of thermal stratification inversion significantly suppresses vertical convection currents, thereby exerting multifaceted impacts on atmospheric environmental dynamics. The presence of TI inhibits air convection, slowing down the diffusion of soot and air pollutants, making atmospheric pollution worse [13]. In recent years, the use of fossil fuels and the rapid development of transportation have led to an increase in the frequency of TI [11]. Although it has been well documented that the presence of TI increases the risk of cardiovascular and respiratory diseases [14], our understanding of TI remains at the tip of the iceberg. The impact of pollutants affected by TI, such as PM_2.5_, on cognitive function has been confirmed [15]. However, evidence directly linking TI to cognitive trajectories is limited.

To address this knowledge gap, we used a nationally representative sample obtained from China Health and Retirement Longitudinal Study (CHARLS) between 2011–2015. We assessed cognitive trajectories in relation to TI to refine understanding of TI’s potential neurocognitive implications.

## 2. Methods

### 2.1. Study design and participant

China Health and Retirement Longitudinal Study (CHARLS) is a representative, interdisciplinary survey program for the middle-aged and elderly population aged 45 years and above [16]. A total of 17,708 people were surveyed. These samples will be followed up every two to three years. Four rounds of regular questionnaire follow-up surveys have now been conducted. The data can be downloaded at the CHARLS home page at http://charls.pku.edu.cn/en. The CHARLS study received ethical approval from the Biomedical Ethics Review Committee of Peking University (IRB00001052–11015), and all participants providing signed informed consent prior to participation.

Because the 2018 wave changed cognitive items, comparability with earlier waves is limited, we ended up using the data from 2011 (wave 1), 2013 (wave 2) and 2015 (wave 3).

**Fig 1:**
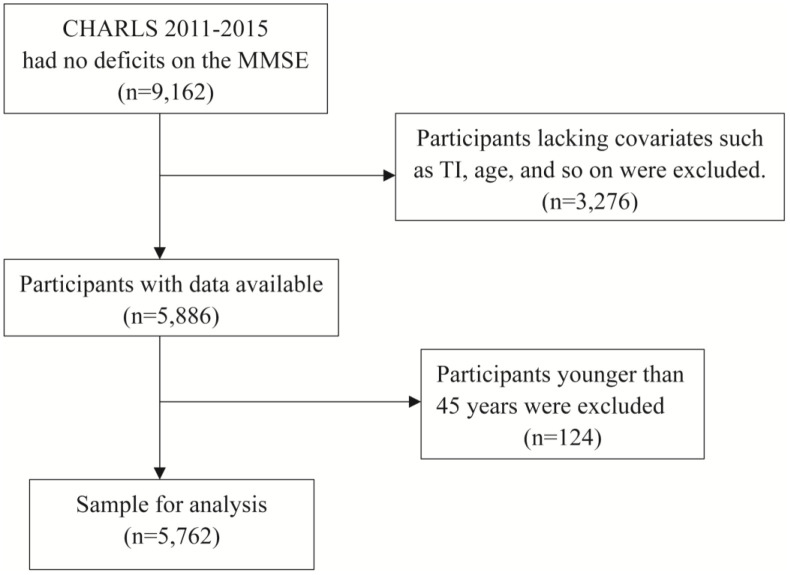
Flow chart of inclusion and exclusion criteria from the CHARLS 2011–2015 database.

A total of 9,162 participants were included in this study, and they fully participated in the three-year surveys conducted in 2011, 2013, and 2015. 3,276 were excluded due to missing information on: TI duration (n = 2,806), age (n = 233), education (n = 1), sex (n = 1), nighttime sleep (n = 29), nap (n = 12), cigarette smoking (n = 1), and chronic disease status (n = 324). Additionally, 124 participants were excluded due to being younger than 45 years old excluded, and ultimately, a total of 5,762 participants were included in this study (Fig 1).

### 2.2. Cognitive assessment

The cognitive function trajectory is fitted based on the total score of cognitive function. The Mini-Mental State Examination (MMSE) was used to assess participants’ cognitive function, with a total score ranging from 0–21, divided into two sections: mental intactness and episodic memory [17,18]. Mental intactness scores range from 0–11 and are divided into orientation (identifying the month, day, and year, the day of the week, and the season of the year, 5), attention (serial subtraction of 7 from 100, up to 5 times, 5), and visuo-construction (figure drawing, 1) [17,18]. Episodic memory scores ranged from 0–10 points and were divided into two parts: immediate recall and delayed recall. In the immediate recall part, respondents were asked to immediately recall 10 Chinese words read by the interviewer, and each correct answer was scored as 1 point for a total of 10 points, and then the respondents recalled them again 4 minutes later, and their scores were recorded as delayed recall scores, with the average of the immediate and delayed recall scores as the episodic memory score [19].

### 2.3. Exposure assessment

The TI data is calculated from NASA’s MERRA-2, version 5.12.4. The dataset, at a resolution of 0.5° × 0.625°, provides temperature data across 42 atmospheric pressure layers. However, given the widespread presence of missing data across multiple atmospheric pressure layers, the TI near the ground has more severe health effects [12], we consequently employed temperature data from the first (100 meters) and second (300 meters) layers for TI calculations. By extracting and comparing the average temperatures of the first and second layers, we can judge the TI situation. If the temperature of the second layer is higher than that of the first layer, the TI is occurring. Otherwise, the city is not experiencing TI [20] (The dataset provides temperature data at four distinct time points within a day. If a TI occurs at any of the four time points, the entire day is deemed to exhibit the TI phenomenon.). After obtaining the TI data, we match the number of TI days to individuals by their addresses. In this study, TI data were grouped using the mean ± standard deviation (SD) method: the calculated mean of TI was 1059.187 with an SD of 341.108, so the low level TI exposure group was defined as TI ≤ (mean – SD), the medium level TI exposure group as (mean – SD) <TI <(mean + SD), and the high level TI exposure group as TI ≥ (mean + SD).

### 2.4. Covariates

Based on previous studies, we finally identified the following variables as covariates [8,21,22], Demographic covariates included age, sex (“male”, “female”). Health behavior covariates included ever/current smoke (“No”, “Yes”), ever/current alcohol (“No”, “Yes”), chronic diseases (“No”, “Yes”), daily sleep time (“≤5”, “5-10”, “≥10”), daily nap time (“0”, “1-29”, “30-90”, “>90 minutes”). Socioeconomic covariates included education level (“Primary school or below”, “Middle school or above”), marital status (“Married and trial separation”, “Divorced, separated, widowed and never married”), residence (“Rural”, “Urban”). Finally, owing to the differences in economic development between the eastern and western parts of China [23], and taking into account the impact of the economic level of different residences [24], we classify them into eastern, midland and western regions in accordance with the geographic division defined by the National Bureau of Statistics of China (NBS) and include them as covariates. This study also took into account the effects of air pollutants such as PM_2.5_, PM_10_, and NO_2_ [25]. The air pollutants datasets are provided by National Tibetan Plateau/ Third Pole Environment Data Center (http://data.tpdc.ac.cn) [26].

### 2.5. Statistical analysis

We used latent class trajectory models (LCTM) to fit the developmental trajectories of cognitive functions in populations. And participants were grouped according to different trajectory class. The core idea of LCTM is to assume the existence of heterogeneity within the population, i.e., that there are differences in trajectories among samples and that they can be classified into different classes [27]. In terms of specific model specifications, the time metric was defined in years, with measurements collected at 0, 2, and 4 years, representing equal 2-year intervals between consecutive assessments. To capture the potential curvilinear trends in cognitive score trajectories over time, a quadratic polynomial order was adopted in the LCTM. The distributional assumptions followed the default normal distribution, and both the number of iterations and convergence criteria used the default settings. Multinomial logistic regression was used to evaluate the relationship between different exposure levels and cognitive function trajectory classes. In order to explore the potential nonlinear relationship between the TI and cognitive functioning, this study used restricted cubic spline (RCS) and determined the number of inflection points based on the Akaike information criterion (AIC) values, the number of nodes is 4, and the default quantiles of 0.05, 0.35, 0.65, and 0.95 are adopted.

Determining the optimal latent class trajectory model requires comparing the Bayesian Information Criterion (BIC; smaller BIC indicates a better model fit), the Average Posterior Probability (AvePP; The average posterior probability of each class is greater than or equal to 0.7 is considered to be a selectable model), and the proportion of the population in each trajectory class (the proportion of each class is generally required to be no less than 5%). In addition, the interpretability of the model is also taken into account [8].

In sensitivity analyses, because of the large effect of age on cognitive function [28], we excluded those older than 65 years and restricted the analysis to those aged 45–65 years to further observe the effect of TI on cognitive function. Besides, to avoid misclassification bias caused by the original definition of TI—where a day was classified as a TI day if TI occurred at any one of the four daily time points—we revised the definition. Specifically, a day is now defined as a TI day when TI occurs at two or more of the four daily time points, and we re-conducted the analysis based on this revised definition. Since PM_2.5_, PM_10_, and NO_2_ may exert a mediating effect on the cognitive function trajectory groups in TI [25], we analyzed these three types of air pollutants as mediating factors.

All statistical analyses and image visualizations were performed using R Version 4.3.3. The *lcmm* package was used to fit the LCTM. With continuous variables expressed as means ± SD and categorical variables were presented as percentages. All *P*-values less than 0.05 (two-way) were considered statistically significant.

## 3. Results

### 3.1. Descriptive characteristics

At baseline, a total of 5,762 middle-aged and elderly people were included in this study, of which 3,058 (53.07%) were men. All participants aged 45–90 years old (mean age 57.88 years) were enrolled in this research. The mean MMSE score was 12.36 out of 21 (SD = 3.29). 1040(18.05%) participants were exposed to high levels of TI over the 5-year period. 2,401 (41.67%) participants from urban. 3,292 (57.13%) participants had received education only at primary school level and below. Most participants were married. In daily life, the number of people with smoking and drinking habits were 2,496 (43.32%) and 2,102 (36.48%) respectively. There were 3,774 (65.50%) participants with chronic disease. 4,099 (71.14%) participants slept between 5–10 hours at night. Nearly half of the participants had little or no napping habit. In addition, nearly half of the participants were from eastern China. The baseline profile of the participants is shown in Table 1.

**Table 1:** Baseline characteristics of the total sample and of the different trajectory classes.

Variables	Total (n = 5,762)	TRAJECTORY CLASS			
		U-shaped class (n = 1,070)	Slowly decline class (n = 3,263)	N-shaped calss (n = 1,429)	*P*-value^a^
TI exposure level					<0.001
Low	1,136 (19.72)	252 (23.55)	528 (16.18)	356 (24.91)	
Medium	3,586 (62.24)	665 (62.15)	2,095 (64.20)	826 (57.80)	
High	1,040 (18.05)	153 (14.30)	640 (19.61)	247 (17.28)	
Age	57.88 ± 8.38	59.70 ± 8.51	56.42 ± 7.86	59.84 ± 8.78	<0.001
Sex					<0.001
Male	3,058 (53.07)	479 (44.77)	1,897 (58.14)	682 (47.37)	
Female	2,704 (46.93)	591 (55.23)	1,366 (41.86)	747 (52.27)	
Residence					<0.001
Rural	3,361 (58.33)	757 (70.64)	1,636 (50.27)	968 (68.02)	
Urban	2,401 (41.67)	313 (29.36)	1,627 (49.73)	461 (31.98)	
Educational level					<0.001
Primary school or below	3,292 (57.13)	840 (78.50)	1,334 (40.88)	1,118 (78.24)	
Middle school or above	2,470 (42.87)	230 (21.50)	1,929 (59.12)	311 (21.76)	
Marry status					<0.001
Married	5,275 (91.55)	943 (88.13)	3,065 (93.93)	1,267 (88.66)	
Separated/Divorced/Widowed/Never married	487 (8.45)	127 (11.87)	198 (6.07)	162 (11.34)	
Ever/current smoke					0.010
No	3,266 (56.68)	629 (58.79)	1,793 (54.95)	844 (59.06)	
Yes	2,496 (43.32)	441 (41.21)	1,470 (45.05)	585 (40.94)	
Ever/current alcohol					<0.001
No	3,660 (63.52)	745 (69.63)	1,946 (59.64)	969 (67.81)	
Yes	2,102 (36.48)	325 (30.37)	1,317 (40.36)	460 (32.19)	
Chronic diseases					0.013
No	1,988 (34.50)	373 (34.86)	1,167 (35.76)	448 (31.35)	
Yes	3,774 (65.50)	697 (65.14)	2,096 (64.24)	981 (68.65)	
Daily sleep time (h)					<0.001
≤ 5	1,462 (25.37)	315 (29.44)	712 (21.82)	435 (30.44)	
5-10	4,099 (71.14)	706 (65.98)	2,460 (75.39)	933 (65.29)	
≥ 10	201 (3.49)	49 (4.58)	91 (2.79)	61 (4.27)	
Daily nap time (m)					<0.001
0	2,533 (43.96)	492 (45.98)	1,349 (41.34)	692 (48.43)	
1-29	517 (8.97)	94 (8.79)	301 (9.22)	122 (8.54)	
30-90	2,072 (35.96)	346 (32.34)	1,261 (38.65)	465 (32.54)	
> 90	640 (11.11)	138 (12.90)	352 (10.79)	150 (10.50)	
Region					<0.001
Eastern	2,525 (43.82)	437 (40.84)	1,504 (46.09)	584 (40.87)	
Midland	1,979 (34.35)	375 (35.05)	1,104 (33.83)	500 (34.99)	
Western	1,258 (21.83)	258 (24.11)	655 (20.07)	345 (24.14)	
PM_2.5_(μg/m³)	58.85 ± 18.37	58.64 ± 18.35	59.32 ± 18.37	57.93 ± 18.36	<0.001
PM_10_(μg/m³)	99.21 ± 31.32	98.62 ± 31.33	100.12 ± 31.31	97.58 ± 31.32	<0.001
NO_2_(μg/m³)	31.25 ± 9.96	30.90 ± 9.94	31.66 ± 9.96	30.56 ± 9.96	<0.001
Episodic memory	3.62 ± 1.80	3.18 ± 1.52	4.38 ± 1.55	2.20 ± 1.53	
Mental intactness	8.74 ± 2.26	7.85 ± 2.28	9.93 ± 1.20	6.71 ± 2.32	
MMSE score	12.36 ± 3.29	11.03 ± 2.87	14.31 ± 1.93	8.91 ± 2.67	<0.001

a. *P* values for comparison between groups, obtained from analyses of variance for continuous variables and χ^2^-test for categorical variables.

### 3.2. Cognitive trajectory modeling

**Fig 2:**
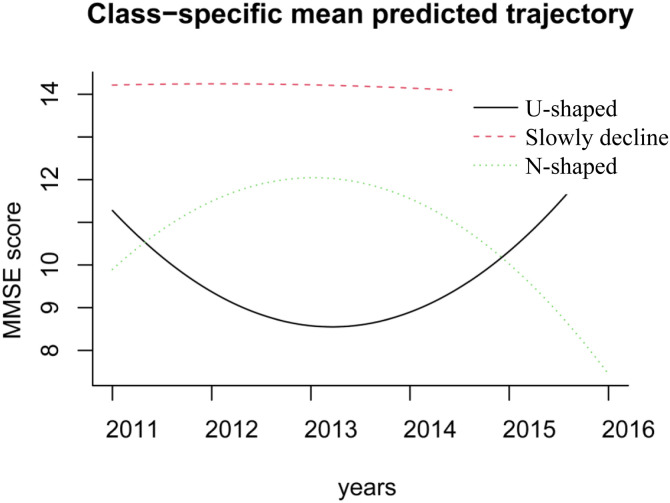
Trajectories of the MMSE scores.

After repeating the iterations, Although 6-class models had lower AIC/BIC, two classes were<5% and one AvePP < 0.7, we selected the 3-class solution for parsimony, class separation (AvePP ≥ 0.76), and interpretability (Table 2). The optimal model divides the population cognitive trajectories into three trajectory class (Fig 2): U-shaped class (decline then improvement) (1,070, 18.57%), Slowly decline class (3,263, 56.63%) and N-shaped class (improvement then decline) (1,429, 24.80%).

**Table 2:** AvePP of Class Assignment and AIC, BIC Statistics of Model Fit.

	AvePP							AIC	BIC	PROPORTION(%)
	G1	G2	G3	G4	G5	G6	G7			
1	1.00							85378.60	85425.21	100
2	0.79	0.86						84734.67	84814.58	58.47/41.53
**3**	**0.76**	**0.80**	**0.76**					**84360.79**	**84474.00**	**18.57/56.63/24.80**
4	0.76	0.72	0.64	0.75				84262.57	84409.06	16.49/23.20/38.11/22.20
5	0.55	0.71	0.71	0.74	0.53			84236.61	84416.41	28.29/20.74/16.94/16.12/17.91
6	0.71	0.74	0.61	0.72	0.52	0.69		84088.51	84301.60	21.05/9.74/36.48/23.65/5.02/4.06
7	0.73	0.62	0.63	0.72	0.71	0.69	0.48	84077.05	84323.44	10.26/34.31/2.07/19.35/3.59/26.85/3.58

### 3.3. Association between TI with trajectories of cognitive function trajectory

Table 3 describes the relationship between cognitive trajectory class and different levels of TI exposure through multinomial logistic regression. Compared with the U-shaped class, higher TI exposure was associated with increased odds of membership in the Slowly decline class. At medium TI exposure, the odds of being classified into the Slowly decline trajectory versus the U-shaped trajectory were 50.4% higher (OR=1.504, 95% CI = 1.264 ~ 1.789, *P* < 0.001), at high exposure, 99.6% higher (OR=1.996, 95% CI = 1.584 ~ 1.517, *P* < 0.001). The statistically significant difference persisted upon adjusting for demographic factors in Model 2. Model 4 adjusting for demographic covariates, health behavior covariates, socioeconomic covariates。 At medium TI exposure, the odds of being classified into the Slowly decline trajectory versus the U-shaped trajectory were 26.9% higher (OR=1.269, 95% CI 1.054 ~ 1.528, *P* = 0.012), at high exposure, 47.8% higher (OR=1.478, 95% CI 1.152 ~ 1.895, *P* = 0.002). No statistically significant association was detected between the N-shaped class and the U-shaped class in any of the multiple model adjustments.

**Table 3:** Multinomial logistic regression: odds of class membership vs U-shaped (reference).

Slowly decline class vs U-shaped class				N-shaped class vs U-shaped class		
Adjustment	OR	95%CI	P	OR	95%CI	P
Model1						
Low	Ref.			Ref.		
Medium	1.504	1.264,1.789	<0.001	0.879	0.726,1.064	0.187
High	1.996	1.584,2.517	<0.001	1.143	0.883,1.480	0.311
Model2						
Low	Ref.			Ref.		
Medium	1.462	1.224,1.745	<0.001	0.883	0.729,1.069	0.203
High	1.967	1.553,2.492	<0.001	1.155	0.892,1.496	0.275
Model3						
Low	Ref.			Ref.		
Medium	1.451	1.215,1.734	<0.001	0.896	0.739,1.085	0.261
High	1.946	1.534,2.468	<0.001	1.172	0.903,1.520	0.233
Model4						
Low	Ref.			Ref.		
Medium	1.269	1.054,1.528	0.012	0.863	0.737,1.083	0.250
High	1.478	1.152,1.895	0.002	1.171	0.901,1.523	0.238

*Note*: Model 1: no adjustment. Model 2: adjust for age, gender. Model 3: adjust for age, gender, chronic diseases, daily sleeping time, daily nap time, alcohol, smoke. Model 4: adjust for age, gender, chronic diseases, daily sleeping time, daily nap time, alcohol, smoke, marital status, education level, residence, region.

**Fig 3:**
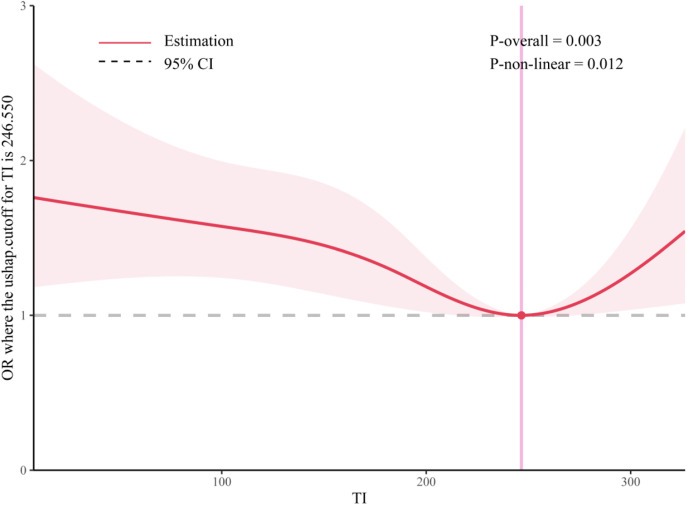
Restricted cubic spline showing the association between TI and cognitive dysfunction.

In addition, according to the results of RCS, the RCS indicated a U-shaped association with a nadir at 246.6 TI days/year (P-nonlinearity = 0.012) (Fig 3).

### 3.4. Sensitivity analysis

After excluding those over 65 years of age, we reconstructed the class trajectory model and performed multinomial logistic regression modeling (Supporting information, S1 Table). At high TI exposure, the odds of being classified into the Slowly decline trajectory versus the U-shaped trajectory were 74.1% higher (OR=1.741, 95% CI = 1.299 ~ 2.334, *P* < 0.001), the odds of being classified into the N-shaped trajectory versus the U-shaped trajectory were 55.5% higher (OR=1.555, 95% CI = 1.128 ~ 2.144, *P* = 0.007). After adjusting the definition of TI (Supporting information, S2 Table), At medium TI exposure, the odds of being classified into the Slowly decline trajectory versus the U-shaped trajectory were 23.8% higher (OR=1.238, 95% CI = 1.022 ~ 1.500, *P* = 0.029), at high exposure, 58.6% higher (OR=1.586, 95% CI = 1.228 ~ 2.047, *P* < 0.001). At high TI exposure, the odds of being classified into the N-shaped trajectory versus the U-shaped trajectory were 51.4% higher (OR=1.514, 95% CI = 1.158 ~ 1.978, *P* = 0.002). Furthermore, in the mediating effect analysis of the three air pollutants on the impact of TI on cognitive function trajectories, none of the mediating effects were significant (Supporting information, S3 Table).

## 4. Discussion

**Fig 4:**
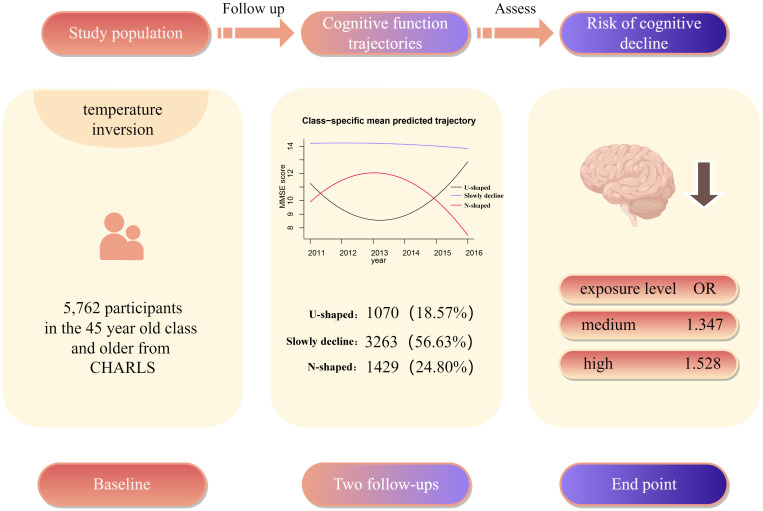
Graphical abstract.

This study analyzed data from 5,762 participants in the CHARLS, and the results revealed significant heterogeneity in cognitive abilities across the population. Additionally, a correlation was identified between the number of TI days and the cognitive abilities of the participants. The RCS indicates that when the number of TI days per year is less than 246.55, there is a continuous decrease in the negative effect of TI on cognition. The downward trend will cease when the number of TI days reaches 246.55 days, at which point the adverse effect of TI on cognitive dysfunction increases (Fig 4).

No statistically significant association was identified between the U-shaped group class and the N-shaped group class. This may be due to the overly similar demographic proportions between the two trajectory group classes. By comparing the demographic composition of the Slowly decline class and the U-shaped class, we found that the population in the Slowly decline class was younger, with a greater proportion of males, and most were from urban areas. Additionally, participants in the Slowly decline class exhibited higher levels of education and a greater prevalence of alcohol consumption.

This finding is in general accordance with the results of previous studies. According to Cattell’s research on cognition, abilities such as memory as well as cognition begin to decline linearly at a young age and are less likely to change in older adults [29]. The results of this study show that higher TI exposure was associated with Slowly decline class membership; given higher education and urban residence in this class at baseline, residual confounding or differential exposure patterns may contribute. This is also consistent with the finding in this study that the Slowly decline class has a higher proportion of urban population. Ethanol, the primary component of alcohol, is a direct neurotoxin that has been demonstrated to cause persistent dementia in sufficient doses [30]. And it is generally accepted that men consume alcohol at a higher rate than women. This may be one of the reasons why the Slow decline class has a higher proportion of males.

We speculate that there may be several reasons why TI has an impact on cognitive function. First, TI affect the dispersion of air pollutants such as black carbon, particulate matter, and nitrogen oxides [11], leading to increased exposure times in the population, which may contribute to cognitive decline in the population [13]. It has been shown that long-term exposure to PM_2.5_ and NO_2_ increases the risk of mild cognitive impairment [15,31]. Animal experiments have found that the activation of microglia plays an important role in the decline of cognitive function [32]. Excessive intake of particulate matter and soluble pollutants leads to microglia activation and neuroinflammation [33]. Dysregulated microglia release neurotoxicity factors such as TNFα, IL-1β, and INF-γ, which can lead to oxidative stress and damage in the brain [34]. This may be the core factor why TI leads to cognitive decline. Second, considering the impact of TI on regional climate, TI can suppress rainfall, when combined with the urban heat island effect, and lead to increased regional temperatures [11]. A study from China has shown that heat exposure may increase the likelihood of dementia in the population [35]. Finally, pollutants suppressed by TI can increase the formation of acid rain and photochemical smog [36], which damages vegetation and water bodies [37]. This weakens their absorption of cognitive-damaging pollutants such as PM_2.5_, thereby further increasing the likelihood of cognitive decline in the population.

This study has the following advantages. Previous studies on cognitive ability have focused on diet, related pollutants, and meteorological conditions [9,10]. However, studies of TI as abnormal meteorological conditions on cognitive function have not yet been found, and studies of TI are relatively rare. Consequently, the present study can contribute to a more comprehensive understanding of the factors influencing cognitive function. In addition, there is a lot of literature on factors that may affect cognitive functioning in humans, but these studies do not take into account the differences that exist between individual cognition. We believe that there are differences in this approach, so we chose the LCTM to categorize the population according to cognitive trajectories in order to make cognitive functioning more realistic [10]. Besides, compared with focusing solely on a single cognitive outcome event at a specific time point, continuously tracking participants’ cognitive status over multiple years is of greater research value.

This study also has the following limitations. Firstly, as the data is derived from the CHARLS, it is constrained by the fact that it does not reflect the circumstances of other regions. Previous studies have shown that there are differences in the cognitive trajectory classes of people in different regions [38,39]. Secondly, only one of the two class of results was statistically significant. The other class did not yield a statistically significant result. However, it was not possible to adjust for this discrepancy due to the fact that the groupings were based on the cognitive trajectories of the population. Moreover, as a large-scale multi-stage sampling cohort, the partial data we used from CHARLS do not include reported survey weights. Further, during the MMSE rating process, the interviewer read aloud three times during the immediate recall portion of the 2013 interview, and this type of questioning was significantly different from the 2011 as well as 2015 interviews. That may have skewed the population’s immediate recall as well as delayed recall scores, which in turn led to a bias in situational memory scores. Although this study only included the scores of the interviewees at the first read-aloud, we were unable to adjust for the effect of delayed recall scores. Finally, for reasons related to individual privacy, the PSU codes in the CHARLS database are only real at the municipal level and above. Therefore, when we match the TI information, we can only match TI information to individuals according to the city level. The TI, as a meteorological condition, may vary more accurately between counties and districts. On the other hand, data matching conducted at the city level may lead to corresponding potential spatial misclassification.

## 5. Conclusion

Increased TI days were associated with higher odds of membership in a non-U-shaped trajectory. Thus, actively promoting relevant environmental protection policies to reduce TI events may support healthier cognitive aging.

## Supporting information

S1 Table Multinomial logistic regression: odds of class membership (with U-shaped as the Reference Group) after excluding participants aged over 65 years. (DOCX)

S2 Table Multinomial logistic regression: odds of class membership (with U-shaped as the Reference Group) after adjusting for the definition of TI. (DOCX)

S3 Table Results of mediating effect of air pollutants between TI and cognitive function trajectories. (DOCX)

## Abbreviations

CHARLS: China Health and Retirement Longitudinal Study
LCTM: latent class trajectory model
RCS: restricted cubic spline
MMSE: mini-mental State Examination
TI: thermal inversion
