# Supplementary material for: Association between thermal inversion and cognitive trajectories among middle-aged and older adults in CHARLS: A latent class trajectory analysis
Source: PLoS One. 2025 Nov 11;20(11):e0335902. doi: 10.1371/journal.pone.0335902 (PMC12604796; doi:10.1371/journal.pone.0335902)
Supplement: S1 Table — (DOCX) [file pone.0335902.s001.docx]

Table S1. Multinomial logistic regression: odds of class membership (with U-shaped as the Reference Group) after excluding participants aged over 65 years

Table S1. Multinomial logistic regression: odds of class membership (with U-shaped as the Reference Group) after excluding participants aged over 65 years

| Slowly decline group vs U-shaped group | | | |  | N-shaped group vs U-shaped group | | | | |
| --- | --- | --- | --- | --- | --- | --- | --- | --- | --- |
| Adjustment | OR | 95%CI | *P* |  | OR | | 95%CI | | *P* |
| Model1 | | | | | | | | | |
| Low | Ref. | | | | | Ref. | | | |
| Medium | 1.418 | 1.172,1.715 | ＜0.001 | | 0.892 | | 0.714,1.115 | 0.316 | |
| High | 2.168 | 1.648,2.853 | ＜0.001 | | 1.527 | | 1.114,2.094 | 0.009 | |
| Model2 | | | | | | | | | |
| Low | Ref. | | | | | Ref. | | | |
| Medium | 1.399 | 1.153,1.698 | ＜0.001 | | 0.891 | | 0.713,1.114 | 0.312 | |
| High | 2.180 | 1.651,2.879 | ＜0.001 | | 1.531 | | 1.117,2.099 | 0.008 | |
| Model3 | | | | | | | | | |
| Low | Ref. | | | | | Ref. | | | |
| Medium | 1.387 | 1.142,1.686 | ＜0.001 | | 0.906 | | 0.724,1.134 | 0.390 | |
| High | 2.121 | 1.602,2.809 | ＜0.001 | | 1.550 | | 1.128,2.130 | 0.007 | |
| Model4 | | | | | | | | | |
| Low | Ref. | | | | | Ref. | | | |
| Medium | 1.218 | 0.984,1.504 | 0.071 | | 0.879 | | 0.694,1.115 | 0.289 | |
| High | 1.741 | 1.299,2.334 | ＜0.001 | | 1.555 | | 1.128,2.144 | 0.007 | |

Note: Model 1: no adjustment; Model 2: adjust for age, gender; Model 3: adjust for age, gender, chronic diseases, daily sleeping time, daily nap time, alcohol, smoke; Model 4: adjust for age, gender, chronic diseases, daily sleeping time, daily nap time, alcohol, smoke, marital status, education level, residence, region.
