# Supplementary material for: Association between thermal inversion and cognitive trajectories among middle-aged and older adults in CHARLS: A latent class trajectory analysis
Source: PLoS One. 2025 Nov 11;20(11):e0335902. doi: 10.1371/journal.pone.0335902 (PMC12604796; doi:10.1371/journal.pone.0335902)
Supplement: S2 Table — (DOCX) [file pone.0335902.s002.docx]

Table S2. Multinomial logistic regression: odds of class membership (with U-shaped as the Reference Group) after adjusting for the definition of TI

Table S2. Multinomial logistic regression: odds of class membership (with U-shaped as the Reference Group) after adjusting for the definition of TI

| Slowly decline group vs U-shaped group | | | |  | N-shaped group vs U-shaped group | | | | |
| --- | --- | --- | --- | --- | --- | --- | --- | --- | --- |
| Adjustment | OR | 95%CI | P |  | OR | | 95%CI | | P |
| Model1 | | | | | | | | | |
| Low | Ref. | | | | | Ref. | | | |
| Medium | 1.422 | 1.214,1.667 | <0.001 | | 0.886 | | 0.741,1.059 | 0.184 | |
| High | 1.996 | 1.575,2.531 | <0.001 | | 1.478 | | 1.138,1.921 | 0.003 | |
| Model2 | | | | | | | | | |
| Low | Ref. | | | | | Ref. | | | |
| Medium | 1.372 | 1.168,1.613 | <0.001 | | 0.891 | | 0.745,1.064 | 0.204 | |
| High | 1.830 | 1.438,2.330 | <0.001 | | 1.494 | | 1.149,1.943 | 0.003 | |
| Model3 | | | | | | | | | |
| Low | Ref. | | | | | Ref. | | | |
| Medium | 1.374 | 1.166,1.618 | <0.001 | | 0.915 | | 0.764,1.096 | 0.334 | |
| High | 1.828 | 1.432,2.333 | <0.001 | | 1.526 | | 1.170,1.988 | 0.002 | |
| Model4 | | | | | | | | | |
| Low | Ref. | | | | | Ref. | | | |
| Medium | 1.238 | 1.022,1.500 | 0.029 | | 0.861 | | 0.702,1.056 | 0.151 | |
| High | 1.586 | 1.228,2.047 | <0.001 | | 1.514 | | 1.158,1.978 | 0.002 | |

Note: Model 1: no adjustment; Model 2: adjust for age, gender; Model 3: adjust for age, gender, chronic diseases, daily sleeping time, daily nap time, alcohol, smoke; Model 4: adjust for age, gender, chronic diseases, daily sleeping time, daily nap time, alcohol, smoke, marital status, education level, residence, region.
