# Supplementary material for: Association between thermal inversion and cognitive trajectories among middle-aged and older adults in CHARLS: A latent class trajectory analysis
Source: PLoS One. 2025 Nov 11;20(11):e0335902. doi: 10.1371/journal.pone.0335902 (PMC12604796; doi:10.1371/journal.pone.0335902)
Supplement: S3 Table — (DOCX) [file pone.0335902.s003.docx]

Table S3. Results of Mediating Effect of Air Pollutants Between TI and Cognitive Function Trajectories

Table S3. Results of Mediating Effect of Air Pollutants Between TI and Cognitive Function Trajectories

| Variables | Total effect Coefficient  (95% CI) | *P*-value | | | Indirect effect Coefficient  (95% CI) | *P*-value | | | Direct effect Coefficient  (95% CI) | *P*-value | |
| --- | --- | --- | --- | --- | --- | --- | --- | --- | --- | --- | --- |
| PM_2.5_ | 0.041(0.009,0.073) | | 0.016 | -0.013(-0.027,0.001) | | | 0.070 | 0.055(0.019,0.091) | | | 0.003 |
| PM_10_ | 0.041(0.008,0.074) | | 0.015 | -0.013(-0.028,0.002) | | | 0.110 | 0.054(0.018,0.090) | | | 0.004 |
| NO_2_ | 0.042(0.008,0.076) | | 0.015 | -0.002(-0.011,0.007) | | | 0.710 | 0.043(0.009,0.077) | | | 0.014 |
